# STAFF FOCUS GROUP TO INFORM A DYADIC PSYCHOLOGICAL INTERVENTION FOR SHORT-STAYS IN NURSING HOME

**DOI:** 10.1093/geroni/igae098.3724

**Published:** 2024-12-31

**Authors:** Evan Plys, Makenna Law, Morgan Seward, Ana-Maria Vranceanu

**Affiliations:** Massachusetts General Hospital, Boston, Massachusetts, United States; Massachusetts General Hospital, Boston, Massachusetts, United States; Massachusetts General Hospital, Boston, Massachusetts, United States; Massachusetts General Hospital, Boston, Massachusetts, United States

## Abstract

**Background:**

Subacute/post-acute nursing home (NH) stays represent a missed opportunity for psychological intervention with patient-caregiver dyads at a highly stressful time. Existing interventions are not designed specifically for this setting and dyadic population. This study aimed to identify: (1) psychological needs to inform intervention content; and (2) context-specific considerations for implementation.

**Methods:**

We conducted 5 focus groups with 28 NH staff (e.g., leadership, clinical, administrative). Interview guides were iteratively developed and aligned with the Designing for Accelerated Translation of Emerging Innovations (DART) framework. Data were analyzed using hybrid indicative-deductive reflexive thematic analysis to map emergent themes onto the DART framework for intervention development (i.e., demand, user-centered design, and context-sensitive).

**Results:**

A dyadic psychological intervention was identified as filling a gap in care, demonstrating value added. Two themes related to demand: (1) staff need help addressing psychological concerns, and (2) psychological services are lacking for short-stay dyads. Four themes related to user needs: (1) fear and worry related to declining health; (2) emotional responses to changes in independence; (3) impact of psychological distress on rehabilitation progress; and (4) impact of psychological distress on discharge. For context-sensitive approaches, staff provided feedback for intervention delivery (e.g., session length), implementation (e.g., referrals), and the research protocol (e.g., randomization).

**Conclusion:**

Qualitative data was matched with theory and evidence-based skills to develop a dyadic psychological intervention for the NH setting: Building Resilience In SKilled nursing care (BRISK). This presentation will present intervention content, implementation, and protocol considerations to be tested in an upcoming open pilot.

